# Calidad técnica de la reanimación cardiopulmonar tras una intervención formativa y relación con variables antropométricas

**DOI:** 10.23938/ASSN.1156

**Published:** 2026-03-31

**Authors:** David Peña-Otero, Pedro Gil-López, Miguel Ángel Iglesias-Blanco, Sergio Galarreta-Aperte, Piedad Gómez-Torres

**Affiliations:** 1 Servicio Cántabro de Salud Hospital Universitario Marqués de Valdecilla Santander Cantabria España; 2 Instituto de Investigación Sanitaria de Valdecilla (IDIVAL) Grupo de Enfermería Santander Cantabria España; 3 Dirección General de la Guardia Civil Jefatura del Servicio de Sanidad Madrid España; 4 Dirección General de la Guardia Civil Unidad de Seguimiento y Evaluación Madrid España; 5 Universidad de Granada Facultad de Ciencias de la Salud Campus Ceuta. Departamento de Enfermería Ceuta España

**Keywords:** Personal de Primera Respuesta, Capacitación en servicio, Entrenamiento simulado, Composición Corporal, Resucitación Cardiopulmonar, Emergency Responders, In-service Training, Simulation Training, Body Composition, Cardiopulmonary Resuscitation

## Abstract

**Fundamento:**

Dada su capacidad de respuesta inmediata, los efectivos de la Guardia Civil representan un recurso esencial en la reanimación cardiopulmonar extrahospitalaria, especialmente en contextos rurales o de difícil acceso. Este estudio tiene como objetivo evaluar la calidad técnica de reanimación cardiopulmonar tras una intervención formativa y su relación con las variables antropométricas.

**Material y métodos:**

Estudio cuasiexperimental con medidas repetidas, sin grupo control, en el que participaron 41 efectivos de la Sección de Intervención Rápida de la Guardia Civil. Se recogieron variables sociodemográficas, antropométricas, y de calidad de la reanimación cardiopulmonar, incluyendo el total score (0-100 puntos) compuesto por *compression score*, *flow fraction* y *ventilation score*. La intervención en simulación (maniquí) se basó en las directrices del Consejo Europeo de Resucitación.

**Resultados:**

Solo una participante era mujer. La mediana de IMC fue de 27,4 kg/m² y el 70,7% presentó un porcentaje de grasa corporal ≥25%. Tras la intervención, se observaron mejoras estadísticamente significativas en el *total score* mediano (64 a 93 puntos), el porcentaje de ventilaciones correctas (33 a 90%), la relación compresión/ventilación (12,2 a 46,3%) y la velocidad media de compresiones (134 a 109/min). La estatura mostró una asociación negativa con el desempeño post-intervención en el modelo ajustado (*β*= -1,024; p=0,001).

**Conclusiones:**

La intervención mejoró significativamente la calidad de la reanimación cardiopulmonar. La estatura influyó negativamente en el rendimiento mientras que la proporción de grasa corporal no se asoció con el desempeño. Las asociaciones con variables antropométricas deben interpretarse con cautela.

## Abstract (en)

**Background:**

Owing to their capacity for rapid response, members of the Spanish Guardia Civil constitute a key resource for out-of-hospital cardiopulmonary resuscitation, particularly in rural or hard-to-reach areas. This study aimed to evaluate cardiopulmonary resuscitation technical quality following a training intervention and to examine its association with anthropometric variables.

**Methods:**

A quasi-experimental study with repeated measures and no control group was conducted in 41 members of the Rapid Intervention Section. Sociodemographic, anthropometric, and cardiopulmonary resuscitation quality variables were collected, including a composite total score (0-100 points) derived from compression quality, chest compression fraction, and ventilation performance. The simulation-based intervention, performed using a manikin, followed European Resuscitation Council guidelines.

**Results:**

Only one participant was a woman. Median BMI was 27.4 kg/m², and 70.7% of participants had a body fat percentage ≥25%. Post-intervention, significant improvements were observed in median total score (64 to 93 points), proportion of correct ventilations (33% to 90%), compression/ventilation ratio (12.2% to 46.3%), and mean compression rate (134 to 109/min). Height was negatively associated with post-intervention performance (β = -1.024; p = 0.001).

**Conclusions:**

The training intervention significantly improved cardiopulmonary resuscitation quality. Height was inversely associated with performance, whereas body fat percentage showed no significant association. These findings should be interpreted with caution given the study design limitations.

## INTRODUCCIÓN

La reanimación cardiopulmonar (RCP) constituye un pilar esencial de la cadena de supervivencia frente a una parada cardiorrespiratoria (PCR). Factores como la profundidad y frecuencia de las compresiones, la descompresión torácica y la mínima interrupción son determinantes para una perfusión cardiaca y cerebral efectiva, aumentando la supervivencia con buena capacidad neurológica^1^^,^^2^.

Además de las condiciones del entorno y de la víctima, la calidad de la RCP se ve influida por las condiciones físicas del reanimador influyen en la calidad de la RCP^3^, ya que variables antropométricas como el índice de masa corporal (IMC), la masa muscular o la estatura se han relacionado con el rendimiento físico^4^^-^^6^. El rendimiento físico puede condicionar la capacidad de mantener la calidad de las compresiones torácicas, especialmente en escenarios prolongados o de alta exigencia, donde la fatiga es un factor limitante^7^. Este aspecto cobra mayor relevancia en reanimadores no sanitarios sin entrenamiento específico o con escasa experiencia práctica, lo que puede relacionarse con una mayor variabilidad en la calidad de la RCP y con una peor capacidad para mantenerla cuando aparece fatiga. Desde un punto de vista biomecánico, la calidad de las compresiones torácicas depende de generar fuerza suficiente con un vector lo más vertical posible, manteniendo la alineación hombros-esternón y un ángulo de brazos estable, a la vez que se garantiza el retroceso completo^8^. En estudios de simulación, la antropometría del reanimador (estatura, peso e IMC) se ha asociado con componentes específicos de la técnica: un mayor tamaño corporal tiende a facilitar la profundidad, pero puede acompañarse de peor cumplimiento del retroceso, y los perfiles extremos pueden presentar un rendimiento global inferior^9^^,^^10^. Además, la postura del reanimador y el ángulo del brazo modifican de forma relevante la calidad de las compresiones, observándose mejores resultados con un ángulo próximo a 90° y con ajustes ergonómicos (como elevarse) cuando la superficie dificulta la alineación^8^. En este marco, una mayor estatura/envergadura podría dificultar mantener de forma sostenida un vector de fuerza estrictamente vertical en posición arrodillada o en superficies no óptimas, afectando al control técnico global, especialmente cuando la maniobra se prolonga o se evalúan métricas compuestas^11^.

Pero también el nivel de conocimientos de la persona reanimadora influye: la formación en soporte vital básico (SVB) mejora sustancialmente la calidad técnica de la RCP, mientras que la ausencia de actualización periódica conlleva una pérdida progresiva del rendimiento^8^^,^^10^. Puesto que cualquier persona puede actuar como primer interviniente, la formación en RCP es clave, y debería ser potenciada por todas las entidades e instituciones presentes en la comunidad^12^.

En este contexto, las Fuerzas y Cuerpos de Seguridad del Estado (FCSE) representan un recurso clave como primeros intervinientes debido a su presencia estratégica en la vía pública y su capacidad de movilización inmediata^13^. Su papel puede ser especialmente determinante en entornos rurales o con tiempos de respuesta prolongados, donde su actuación puede adelantarse a la llegada de los servicios sanitarios^14^. Entre estas unidades, la Sección de Intervención Rápida (SIR) de la Guardia Civil Española desempeña funciones en provincias sin presencia de Unidades de Seguridad Ciudadana (USECI). Debido a su preparación táctica y su despliegue rápido, los efectivos de la SIR pueden verse implicados en situaciones de PCR en escenarios hostiles, donde su intervención puede ser decisiva para la supervivencia de la víctima.

En España, la participación de fuerzas policiales como primeros intervinientes se ha evaluado principalmente mediante desenlaces asistenciales (por ejemplo, registros de supervivencia^15^) y tiempos de respuesta en un contexto en el que las guías contemporáneas recomiendan integrar y activar primeros intervinientes para reducir el tiempo hasta el inicio de la RCP y la desfibrilación^16^. En otros contextos, los estudios en policías han sido con frecuencia transversales y se han centrado en métricas de compresión registradas con simuladores o en evaluaciones puntuales del desempeño^17^. Por tanto, persiste un vacío aplicable a unidades operativas no sanitarias -como la SIR- en relación a cuantificar el cambio de la calidad técnica de la RCP tras formación y a explorar si las variables antropométricas del reanimador se asocian al rendimiento.

Por tanto, el objetivo del estudio es evaluar el efecto de una intervención formativa en efectivos de la SIR sobre la calidad técnica de las maniobras de RCP en maniquí, analizando su relación con las variables antropométricas.

## MÉTODOS

*Diseño del estudio.* Se realizó un estudio cuasiexperimental con medidas repetidas (pre-post), sin grupo control, mediante una intervención formativa en RCP dirigida a todo el personal operativo de la SIR de Lugo (España) (SIR-GCL), y realizada el 14 de febrero de 2025. De forma complementaria, se realizó un estudio transversal exploratorio para examinar la relación entre variables antropométricas y los indicadores de calidad de la RCP obtenidos en la evaluación. Este estudio ha seguido las recomendaciones *TREND statement*.

*Participantes.* El reclutamiento se realizó dos semanas antes de la intervención y los participantes podían abandonar el estudio si así lo consideraban. Todos los participantes contaban con formación previa en RCP, reglada o no. Se consideró formación reglada a la formación periódica incluida en el plan formativo del grupo operativo, con periodicidad anual e impartida por un enfermero instructor del Plan Nacional de RCP de la Sociedad Española de Medicina Intensiva, Crítica y Unidades Coronarias (PNRCP-SEMICYUC) y, a efectos de este estudio, que había sido realizada en los 24 meses previos a la intervención (periodo de validez del título según el PNRCP-SEMICYUC en el momento de la intervención). Se consideró formación no reglada a la formación sanitaria táctica recibida dentro del plan formativo, sin un programa estandarizado de SVB ni impartida por instructores acreditados.

*Intervención*. La intervención fue llevada a cabo por dos miembros del equipo investigador- enfermeros e instructores del PNRCP-SEMICYUC- con más de 15 años de experiencia docente en el campo. La evaluación de la RCP se realizó mediante simulación con maniquíes de entrenamiento *Little Anne* QCPR® (Laerdal Medical AS®) con retroalimentación objetiva, registrándose automáticamente los indicadores de calidad a través del software QCPR Instructor®. Cada participante realizó una secuencia estandarizada de RCP inmediatamente antes y después de la intervención formativa, en condiciones controladas y homogéneas para todos los evaluados. Cada secuencia tuvo una duración de 2 minutos^2^, en línea con las recomendaciones del ERC sobre el tiempo de los ciclos de RCP.

**Figura 1:**
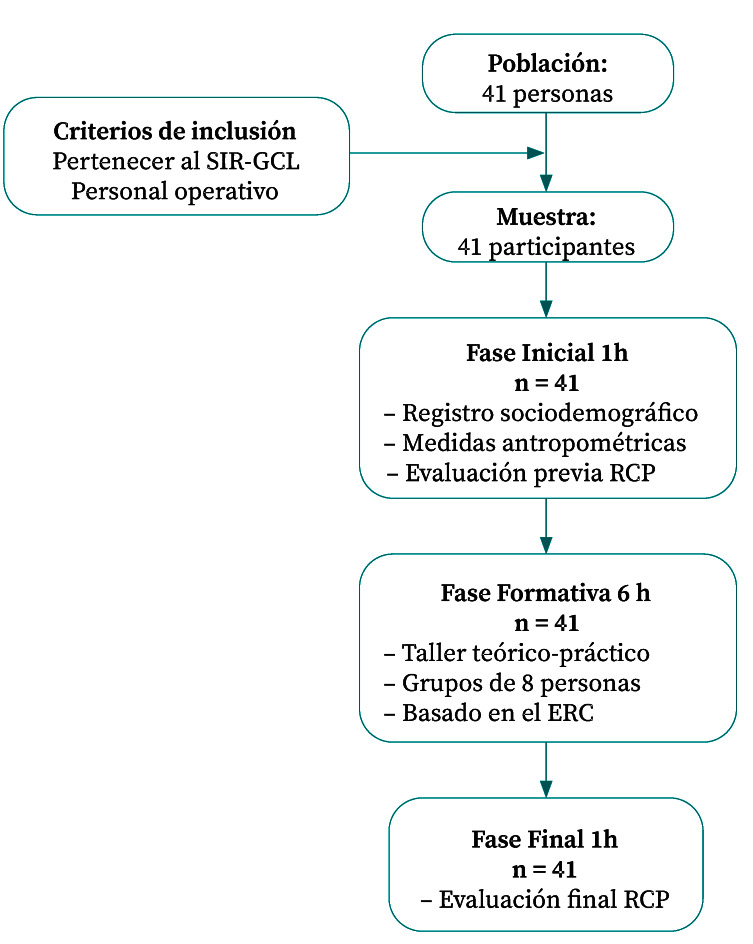
Fases de desarrollo de la intervención.

La intervención tuvo una duración de 8 horas, distribuidas en tres fases (Fig. 1).

SIR-GCL: Sección de Intervención Rápida. Guardia Civil de Lugo; RCP: Reanimación Cardio-Pulmonar; ERC: European Resuscitation Council (Consejo Europeo de Resucitación)

- En la fase inicial (1 hora) se realizaron el registro de variables sociodemográficas, las mediciones antropométricas mediante una báscula de bioimpedancia (*Body Composition Monitor* OMRON® BF511) y un tallímetro de pared, y la evaluación previa de RCP.
- La fase formativa (6 h) consistió en un taller teórico-práctico basado en las directrices del Consejo Europeo de Resucitación (*European Resuscitation Council* - ERC), centrado en la ejecución de compresiones torácicas de alta calidad (frecuencia, profundidad, retroceso completo y minimización de interrupciones) y ventilación boca-a-boca con barrera, trabajando la coordinación de ciclos compresión-ventilación. Los participantes se organizaron en grupos de ocho personas y realizaron práctica supervisada con retroalimentación en tiempo real proporcionada por el software *QCPR Instructor*®, con corrección individualizada por los instructores durante los ejercicios. No se incluyó entrenamiento con desfibrilador. Cada participante fue identificado mediante un código único, ciego para el equipo investigador, que permitió mantener la trazabilidad.
- La evaluación de la calidad de la RCP (1 hora) se realizó inmediatamente después de la intervención formativa.

*Variables de estudio*. Se recogieron distintos tipos de variables independientes: *sociodemográficas:* edad (años), sexo (hombre/mujer) y existencia de formación previa en RCP (reglada/no reglada); y *antropométricas*: peso (kg), estatura (cm), índice de masa corporal (kg/m²), grasa corporal (%), masa muscular (%), metabolismo basal (kcal), flujo espiratorio máximo (FEM), cooximetría en aire espirado (ppm) y tiempo diario dedicado a ejercicio físico (minutos/día). Todas ellas se registraron entre las 08:30 y las 09:30 horas.

Las variables de resultado en relación a la calidad de la RCP fueron: relación compresión-ventilación (correcta/incorrecta), ventilaciones correctas (%), profundidad de las compresiones (correcta/incorrecta), descompresión torácica (completa/incompleta), flujo espiratorio correcto, velocidad media de compresiones (número por minuto), y puntuación total (*total score*: 0-100; rango óptimo: 86-97), proporcionada por el software QCPR a través del *compression score* (profundidad, y frecuencia de compresiones por ciclo o velocidad, cuyo rango óptimo es 106-111 compresiones/minuto), el *flow fraction* y el *ventilation score* (volumen, frecuencia y tiempo inspiratorio de las ventilaciones). 

*Consideraciones éticas.* El estudio fue aprobado por el Servicio de Asistencia Sanitaria de la Dirección General de la Guardia Civil y la Comandancia de la Guardia Civil de Lugo, los organismos militares competentes en la evaluación y autorización de proyectos de investigación (Ref.: MACM/icm. Nº de salida: 19400), y se siguieron las normativas nacionales e internacionales sobre investigación clínica. La participación fue voluntaria, previa información y firma de consentimiento informado. 

### Análisis estadístico

El análisis de datos y resultados fue realizado con el software RStudio v2024.9.0 para Mac por dos miembros del equipo investigador no relacionados con la intervención, que trabajaron con datos anónimos.

Para el análisis descriptivo se utilizaron medidas de tendencia central y dispersión para las variables continuas, así como frecuencias absolutas y relativas para las categóricas. Para evaluar los cambios en la calidad de la RCP se aplicó la prueba de rangos con signo de Wilcoxon, al tratarse de datos apareados no paramétricos. Para las comparaciones entre grupos se utilizó la prueba de U de Mann Whitney para dos grupos y la prueba de Kruskal-Wallis para tres o más grupos. Para analizar los cambios en variables categóricas se utilizó la prueba de McNemar. Las correlaciones entre variables cuantitativas se analizaron mediante el coeficiente de correlación de Pearson.

Se calcularon los tamaños del efecto para los cambios observados, empleando la *r* de Wilcoxon en el caso de variables continuas y el coeficiente phi (Φ) de McNemar para variables categóricas. En ambos casos, la magnitud del efecto se interpretó según los puntos de corte convencionales de Cohen en valor absoluto: <0,10 trivial, 0,10-0,29 pequeña, 0,30-0,49 moderada y ≥0,50 grande.

Para explorar la influencia de las variables antropométricas sobre la calidad de la RCP se realizó un modelo de regresión lineal múltiple con las variables que mostraron diferencias pre y post intervención. Para controlar la multicolinealidad entre predictores, se seleccionaron las variables con un VIF (Factor de Inflación de la Varianza) inferior a 2 (Anexo I). Para la variable categórica dicotómica relación compresión/ventilación se ajustaron modelos de regresión logística pre y post intervención, incluyendo como predictores las variables retenidas tras el control de multicolinealidad (VIF < 2): estatura, FEM, cooximetría y minutos de ejercicio diario. El nivel de significación estadística se estableció en *p* < 0,05 en contraste bilateral y se calcularon intervalos de confianza del 95 % (IC95%) para los coeficientes de los modelos. El análisis de la relación entre variables antropométricas y de calidad de la RCP se planteó con carácter exploratorio, con el objetivo de identificar posibles patrones en la población a estudio.

## RESULTADOS

Participaron 41 personas en el estudio, el total de la población elegible, mayoritariamente hombres (98%), con una edad media de 40,2 años (DE: 6,15). No hubo pérdida de participantes en ninguna fase del estudio.

Todos habían recibido formación previa en RCP, el 73,1% de forma reglada. La mediana del IMC fue de 27,4 kg/m² (RIC: 24,7-29,2) y la del porcentaje de grasa corporal fue 26,7 (RIC: 24,0-30,6), superior al 25% en 29 participantes (70,7%). Otros parámetros relevantes fueron: porcentaje mediano de masa muscular 34,3% (RIC: 32,2-35,5); metabolismo basal mediano 1.764 kcal/día (RIC: 1.660-1.869), y ejercicio diario 50 minutos (RIC: 30-60) (Tabla 1).

**Tabla 1:** Características de la muestra

Característica	Descripción
Sexo (hombre), *n(%)*	40 (97,6)
Formación en SVB (reglada), *n(%)*	30 (73,2%)
Características físicas, *mediana (RIC)*	
Estatura	176 (172,5-180)
Peso	83 (76-90)
IMC	27,4 (24,7-29,2)
% grasa	26,7 (24,0-30,6)
% músculo	34,3 (32,2-35,5)
Metabolismo basal	1.764 (1.660-1.869)
FEM	650 (550-710)
Cooximetría	2 (1-3)
Minutos de ejercicio diario	50 (30-60)

RIC: rango intercuartílico; formación SVB reglada: curso periódico del plan formativo del grupo operativo, impartido por enfermeros instructores del PNRCP-SEMICYUC y realizado en los 24 meses previos, en comparación con la formación SVB no reglada: formación sanitaria táctica interna, sin programa estandarizado de SVB ni impartición por instructores acreditados.

Algunas variables antropométricas se correlacionaron entre sí de forma estadísticamente significativa: la edad mostró relación inversa con el metabolismo basal y la estatura de forma directa con el peso y el metabolismo basal. El peso y el IMC mostraron relación directa entre sí y con la masa grasa y el metabolismo basal, y relación inversa con la masa muscular. Los minutos de ejercicio diario correlacionaron negativamente con la estatura y con la cooximetría (Anexo II).

Respecto a la calidad técnica de la RCP, se observaron diferencias estadísticamente significativas entre las mediciones pre-post de la mayoría de parámetros, con tamaños de efecto grandes. Aumentaron el *total score*, la relación compresión/ventilación correcta y el porcentaje de ventilaciones correctas, mientras que la velocidad media de compresiones disminuyó significativamente, entrando en el rango óptimo (Tabla 2).

**Tabla 2:** Calidad de la RCP pre y post intervención

Variable	Estimador	Tamaño del efecto	p
Total score - *mediana (RIC)*			
Pre	64 (35-75)	Φ = 0,818	<0,001
Post	93 (86-97)		
Relación compresiones / ventilaciones - correcta, *n (%)*			
Pre	(12,19)	r = 0,722	0,002
Post	19 (46,34)		
Flujo - *mediana (RIC)*			
Pre	73 (67-89)	Φ = 0,172	0,648
Post	72 (70-75)		
Porcentaje de descompresiones - *mediana (RIC)*			
Pre	100 (95-100)	Φ = 0,244	0,117
Post	00 (100-100)		
Porcentaje de ventilaciones correctas - *mediana (RIC)*			
Pre	33 (0-64)	Φ = 0,710	<0,001
Post	0 (72-96)		
Profundidad de compresiones - *mediana (RIC)*			
Pre	96 (75-100)	Φ = 0,293	0,06
Post	98 (88-100)		
Velocidad media - *mediana (RIC)*			
Pre	134 (124-152)	Φ = 0,813	<0,001
Post	109 (106-111)		

RIC: rango intercuartílico; Φ: estimador de tamaño de efecto de McNemar; r: estimador de tamaño de efecto de Wilcoxon.

**Figura 2:**
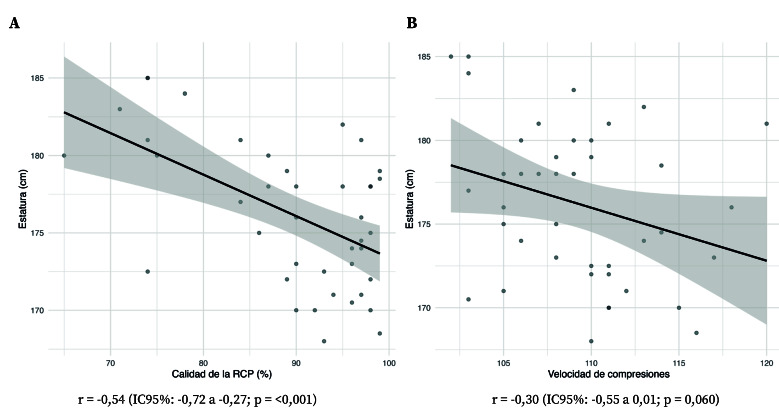
Relación de la calidad de la reanimación cardio-pulmonar con la estatura (A) y con la velocidad de compresiones (B).

En el análisis exploratorio realizado mediante regresión lineal múltiple y regresión logística para estimar las distintas variables de resultado pre y post intervención (Anexo III), solo el modelo de regresión lineal para la puntuación total posterior a la intervención fue significativo (R² ajustado = 0,219, p = 0,011), mostrando una relación negativa con la estatura (β = -1,024; IC95%: -1,644 a -0,404; p = 0,001) tras ajustar por flujo espiratorio máximo, cooximetría y minutos de ejercicio diario. Este resultado sugiere que, a mayor estatura, menor fue el rendimiento global tras la formación (Fig. 2). La estatura también se asoció negativamente con la velocidad media de compresiones post intervención (β = -0,309; IC95%: -0,618 a -0,004; p = 0,049). En el resto de variables, solo se halló una asociación positiva débil entre minutos de ejercicio diario y ventilaciones correctas en la medición previa (β = 0,443; IC95%: 0,011 a 0,875; p = 0,044). Ninguna variable se relacionó con la calidad de la RCP (compresión/ventilación) ni pre ni post intervención (Anexo III).

## DISCUSIÓN

El presente estudio evaluó el efecto de una intervención formativa en RCP sobre la calidad técnica de las maniobras bajo condiciones de simulación realizadas por efectivos de la SIR de la Guardia Civil Española, incorporando el análisis de variables antropométricas del reanimador como posibles moduladores del rendimiento técnico. Los resultados mostraron mejoras significativas en variables clave tras la intervención, especialmente en el *total score*, las ventilaciones correctas, la relación compresión/ventilación adecuada y la velocidad media de compresiones, lo que es consistente con lo documentado previamente sobre el impacto positivo de la formación en soporte vital básico (SVB) en la calidad de la RCP^18^^,^^19^.

El análisis de las variables antropométricas se planteó, ante la limitada evidencia específica en primeros intervinientes no sanitarios, como una exploración inicial en una población operativa, y deben interpretarse como elementos de caracterización de la muestra. Los participantes del estudio presentaron características físicas esperables para una población operativa. Aunque la mediana del IMC fue de 27,4 kg/m², sobrepeso según la OMS, estudios recientes como el de Gažarová y col^20^ sugieren que este valor debe interpretarse con precaución y siempre en conjunto con otros indicadores como el porcentaje de grasa corporal, la masa muscular y el perímetro abdominal. Esto permitiría calcular el *Weight-Adjusted Waist Index* (WWI) como medida más precisa de composición corporal^20^. Los porcentajes de masa grasa y muscular de los participantes de nuestro estudio, a pesar de provenir de una población activa (mediana de 50 minutos diarios de ejercicio), reflejan un perfil de grasa visceral elevado, lo que podría ser relevante en condiciones de alta demanda (como fatiga o en escenarios más exigentes). Esto sugiere la necesidad de determinar adecuadamente la composición corporal. En el presente estudio no se observaron asociaciones estadísticamente significativas entre las variables antropométricas analizadas y la calidad de la RCP, salvo la relación negativa de la estatura con el rendimiento post-intervención. Por ello, medidas como incorporar evaluaciones físicas específicas o planes individualizados de acondicionamiento deberían interpretarse como hipótesis programáticas a explorar y requerirían evaluación adicional antes de generalizar su aplicación dentro de programas como, por ejemplo, el Plan Integral de Promoción del Deporte en la Guardia Civil (PIDEGUCI).

El porcentaje de grasa hallado (26,7%), a su vez, superó el valor de referencia para hombres físicamente activos (10-22%)^21^, y puede estar asociado a una menor masa magra, así como a una mayor carga cardiovascular durante maniobras de RCP mantenidas. Bibl y col encontraron, en simulación pediátrica, que un IMC más bajo se asocia con mayores puntuaciones globales de compresión, mientras que un IMC más alto se relaciona con una mayor tendencia a exceder la profundidad recomendada y a no alcanzar un retroceso completo, especialmente en el maniquí adolescente^10^. En adultos, se han descrito asociaciones positivas entre IMC y probabilidad de realizar RCP de alta calidad (o mayor profundidad)^22^, pero también penalizaciones en el retroceso a medida que aumentan altura, peso e IMC^9^, mientras que otros estudios no encuentran diferencias relevantes por IMC en parámetros de calidad en maniquí^23^^,^^24^.

Aunque no se observó relación entre la edad y las variables antropométricas, sí se encontraron correlaciones entre la estatura y otras variables físicas: positiva con el peso -esperable desde un punto de vista morfológico- y negativa con el ejercicio físico; aunque estadísticamente significativas, estas asociaciones biomecánicas no tienen una implicación clínica directa en este estudio.

Sin embargo, una mayor estatura se asoció con una menor puntuación total tras la formación, relación que se mantuvo tras el ajuste del modelo, junto con una asociación negativa con la velocidad media de compresiones cercana al umbral de significación. La literatura al respecto tampoco es uniforme: algunos estudios asocian mayor estatura o longitud de extremidades con mejor desempeño en parámetros de compresión^25^, mientras que en otros se han descrito penalizaciones en componentes específicos, como retroceso incompleto^9^^,^^25^. Una explicación plausible a la correlación negativa es que, en la posición de arrodillado, una mayor envergadura pueda dificultar mantener un vector de fuerza estrictamente vertical y un ángulo de brazos estable, lo que podría repercutir en el control global de la técnica^8^. En conjunto, la evidencia sugiere que la antropometría no determina de forma uniforme la calidad de la RCP, sino que puede modular componentes específicos en función de la ergonomía impuesta por el escenario; por ello, en reanimadores de mayor talla resulta razonable enfatizar durante el entrenamiento ajustes posturales y del entorno (p. ej., alineación de hombros, posición de rodillas y superficie/altura) para minimizar posibles desventajas biomecánicas^26^. En conjunto, estas discrepancias apuntan a un efecto dependiente del contexto y del componente de calidad evaluado. Desde un punto de vista práctico, estos resultados sugieren la pertinencia de reforzar durante el entrenamiento ajustes posturales y del entorno (como alineación de hombros, posición de rodillas y elección de superficie/altura) para minimizar posibles desventajas biomecánicas en reanimadores de mayor talla.

La intervención formativa mejoró variables técnicas susceptibles de entrenamiento. La puntuación total aumentó tras la formación con un tamaño del efecto grande, lo que sugiere una mayor adherencia global a los componentes de calidad evaluados. Dentro de dicha puntuación, se registraron mejoras en el *ventilation score* -en particular, en el porcentaje de ventilaciones correctas- y en el *compression score*, con una disminución de la velocidad de compresión y una mejora de la relación compresión/ventilación. En términos prácticos, estos cambios implican menos ventilaciones ineficaces y un patrón de compresiones más controlado, lo que podría favorecer la efectividad de las maniobras en un escenario real. Estas mejoras concuerdan con otros estudios^1^^,^^27^ que relacionan la formación estructurada y la percepción del ritmo con la mejora de la técnica en RCP. 

Por el contrario, el flujo pulmonar no presentó cambios significativos, lo que resulta esperable ya que este parámetro no depende directamente del aprendizaje técnico, sino de capacidades funcionales individuales como la eficiencia ventilatoria y la fatiga, influenciadas por la condición física^28^. No se observaron correlaciones significativas entre el flujo espiratorio máximo y las variables antropométricas, aunque sí se identificó una correlación negativa entre la cooximetría y el ejercicio físico diario, compatible con una mejor capacidad ventilatoria en individuos más activos^29^. Tampoco se detectaron diferencias post intervención en relación con la profundidad de compresiones, posiblemente debido a que el nivel técnico previo a la formación ya era adecuado (96% de cumplimiento).

La mejora observada incluso en participantes con buen desempeño basal refuerza la necesidad de formación continuada para mantener y actualizar regularmente las habilidades en SVB y evitar la pérdida progresiva de calidad^13^^,^^19^. El proyecto liderado por la Guardia Civil orientado a proporcionar formación de calidad a todo el personal, adaptada a las necesidades del servicio, se alinea con esta necesidad, y sus resultados apoyan la utilidad de programas formativos con retroalimentación inmediata en cuerpos de seguridad y primeros intervinientes no sanitarios, con un perfil similar al estudiado, que por su despliegue estratégico en zonas rurales pueden desempeñar un papel clave en la cadena de supervivencia^14^^,^^30^^,^^31^.

Sin embargo, al tratarse de una evaluación puntual en simulación y en una población muy específica (efectivos de la Guardia Civil), estos hallazgos no son extrapolables automáticamente a la población general ni a personal sanitario, y deben interpretarse en el marco de primeras respuestas no sanitarias, con reanimadores de características y entrenamiento comparables. Porque a pesar de que la RCP es altamente tiempo-dependiente y la intervención rápida de los primeros testigos es clave, la tasa de inicio de maniobras de RCP por testigos varía considerablemente entre países^31^, y en España, es inferior al 25%^32^ de las más de 25.000 PCR extrahospitalarias anuales.

Este estudio presenta algunas limitaciones metodológicas que deben tenerse en cuenta. Una de las principales fue la ausencia de un grupo control, decisión fundamentada en criterios éticos, dado que los participantes forman parte de un cuerpo de seguridad y se consideró inapropiado restringir el acceso a una formación potencialmente vital. Por este motivo, se ofreció la intervención a todos los voluntarios, priorizando la equidad y evitando conflictos derivados de la exclusión. A su vez, el análisis estadístico debe interpretarse con cautela, ya que el pequeño tamaño muestral (a pesar de haber participado la unidad SIR-GCL al completo) puede conllevar riesgo de sobreajuste en los modelos multivariables; por este motivo, los modelos de regresión se plantearon con carácter exploratorio, sin finalidad predictiva. Sin embargo, la falta de un grupo comparativo, añadida al tamaño muestral, limita la inferencia causal y la capacidad de atribuir de manera concluyente los cambios observados exclusivamente a la intervención. Para mitigar esta debilidad, se aplicaron estrategias de control interno, como el enmascaramiento parcial de las variables que serían evaluadas en la segunda medición, lo que permitió reducir el sesgo de expectativa y los efectos de aprendizaje derivados de la repetición de pruebas.

Las propias características de la muestra limitan la validez externa del estudio. Solo había una mujer entre los participantes, lo que introduce un sesgo de género por la propia composición de la unidad y limita la extrapolación de los hallazgos a mujeres. La edad media del grupo (40,2 años) refleja un colectivo profesional con experiencia, lo que pudo influir en el desempeño previo y posterior a la intervención y limita la extrapolación de resultados a poblaciones más jóvenes o sin experiencia. Futuras investigaciones deberían explorar la interacción entre composición corporal, fatiga y rendimiento técnico en escenarios prolongados y con mayor carga física, para avanzar hacia una formación más precisa, eficaz y realista.

El diseño del estudio -con evaluación puntual en un entorno simulado- es otra limitación. La mejora en indicadores técnicos observada en simuladores no permite inferir impacto clínico, ni tampoco reflejar el rendimiento sostenido en escenarios reales de parada cardiaca con estrés, fatiga o condiciones climáticas adversas, aspectos que merecen atención en investigaciones futuras. Además, el conocimiento de estar siendo evaluados podría haber influido en el desempeño de los participantes (efecto Hawthorne), incrementando la adherencia a la técnica recomendada durante las mediciones.

En conclusión, los resultados obtenidos indican que, en esta cohorte de personal operativo de la Guardia Civil y en condiciones de simulación, la intervención formativa se asoció con mejoras inmediatas en indicadores objetivos de calidad de la RCP, como la puntuación global, las ventilaciones correctas y la relación compresión/ventilación, y sugiere la conveniencia de considerar el perfil antropométrico del reanimador durante el entrenamiento. La estatura mostró una influencia negativa sobre el rendimiento post-intervención, lo que sugiere un posible impacto biomecánico en la ejecución de la RCP. No se identificó una relación directa entre la grasa corporal y la calidad técnica de la RCP.

Estos resultados respaldan la formación periódica en RCP adaptada a las características físicas del reanimador y destacan la necesidad de futuras investigaciones que exploren el efecto de la fatiga y la composición corporal sobre el desempeño prolongado.

## Data Availability

Se encuentran disponibles bajo petición al autor de correspondencia.

## ANEXO I

**Table:** Cálculo de Factor de Inflación de la Varianza (Variance Inflation Factor)

Variable	VIF paso 1	VIF paso 2
Estatura	8,76	1,2
Peso	128,78	-
IMC	49,55	-
Porc_grasa	46,45	-
Porc_musc	50,59	-
Metab_basal	94,85	-
FEM	1,25	1,12
Cooximetría	1,32	1,21
Ejercicio día minutos	1,60	1,28

VIF: variance inflation factor (factor de inflación de la varianza)

## ANEXO II

**Table:** Correlación entre las variables de características físicas

Variables relacionadas	r (IC95%)	p
*Edad*		
Peso	-0,196 (-0,475 a 0,119)	0,220
IMC	-0,074 (-0,373 a 0,239)	0,646
% de grasa	-0,019 (-0,324 a 0,291)	0,908
% muscular	-0,164 (-0,449 a 0,151)	0,305
Metabolismo basal	-0,315 (-0,568 a -0,008)	0,045
Ejercicio diario	0,211 (-0,103 a 0,487)	0,185
*Estatura*		
Peso	0,439 (0,152 a 0,658)	0,004
IMC	0,056 (-0,256 a 0,358)	0,726
% de grasa	0,056 (-0,256 a 0,357)	0,729
% muscular	-0,070 (-0,370 a 0,242)	0,661
Metabolismo basal	0,429 (0,14 a 0,651)	0,005
Ejercicio diario	-0,334 (-0,582 a -0,03)	0,033
*Peso*		
IMC	0,909 (0,835 a 0,951)	<0,001
% de grasa	0,703 (0,504 a 0,831)	<0,001
% muscular	-0,603 (-0,768 a -0,362)	<0,001
Metabolismo basal	0,976 (0,954 a 0,987)	<0,001
Ejercicio diario	-0,283 (-0,543 a 0,027)	0,073
*IMC*		
% de grasa	0,77 (0,605 a 0,871)	<0,001
% muscular	-0,646 (-0,795 a -0,422)	<0,001
Metabolismo basal	0,890 (0,802 a 0,94)	<0,001
Ejercicio diario	-0,14 (-0,429 a 0,175)	0,383
*FEM*		
Cooximetría	0,247 (-0,064 a 0,516)	0,118
IMC	0,023 (-0,287 a 0,328)	0,887
Estatura	0,237 (-0,076 a 0,507)	0,136
Edad	-0,147 (-0,435 a 0,168)	0,358
Peso	0,083 (-0,231 a 0,381)	0,606
% grasa	-0,116 (-0,409 a 0,198)	0,469
% masa muscular	0,136 (-0,179 a 0,425)	0,396
Ejercicio diario	-0,141 (-0,430 a 0,173)	0,377
*Cooximetría*		
Minutos de ejercicio diario	-0,343 (-0,588 a -0,03)	0,028
Edad	-0,040 (-0,343 a 0,270)	0,801
Estatura	0,028 (-0,282 a 0,333)	0,862
Peso	-0,040 (-0,343 a 0,271)	0,804
IMC	-0,066 (-0,366 a 0,247)	0,684

r: coeficiente de correlación de Pearson; IC95%: intervalo de confianza al 95%; IMC: índice de masa corporal.

## ANEXO III

**Table:** Resumen de modelos de regresión lineal y regresión logística para la relación compresión/ventilación (correcta/incorrecta)

Variable	Regresión lineal		
	Estimador β (IC95%)	p	R^2^ ajustado (p)
*Total score previo*			
Estatura	0,218 (-1,518 a 2,018)	0,806	0,003 (0,402)
Flujo espiratorio máximo	-0,008 (-0,086 a 0,070)	0,830	
Cooximetría	1,306 (-0,021 a 2,633)	0,053	
Minutos de ejercicio diario	0,147 (-0,158 a 0,453)	0,147	
*Total score posterior*			
Estatura	-1,024 (-1,644 a -0,404)	0,001	0,219 (0,011)
Flujo espiratorio máximo	-0,009 (-0,036 a 0,017)	0,472	
Cooximetría	0,049 (-0,407 a 0,506)	0,826	
Minutos de ejercicio diario	-0,001 (-0,106 a 0,104)	0,980	
*Velocidad media previa*			
Estatura	-0,576 (-2,123 a 0,969)	0,454	-0,070 (0,849)
Flujo espiratorio máximo	0,013 (-0,054 a 0,080)	0,696	
Cooximetría	-0,298 (-1,439 a 0,841)	0,598	
Minutos de ejercicio diario	0,039 (-0,223 a 0,302)	0,762	
*Velocidad media posterior*			
Estatura	-0,309 (-0,618 a -0,004)	0,049	0,108 (0,086)
Flujo espiratorio máximo	0,012 (-0,007 a 0,026)	0,062	
Cooximetría	0,065 ( -0,162 a 0,29)	0,566	
Minutos de ejercicio diario	0,017 ( -0,035 a 0,07)	0,503	
*Porcentaje de compresiones con profundidad correcta previo*			
Estatura	0,082 (-1,574 a 1,738)	0,92	0,004 (0,399)
Flujo espiratorio máximo	-0,021 (-0,093 a 0,051)	0,554	
Cooximetría	-1,047 (-2,268 a 0,174)	0,090	
Minutos de ejercicio diario	-0,661 (-0,348 a 0,215)	0,636	
*Porcentaje de compresiones con profundidad correcta posterior*			
Estatura	0,310 (-0,779 a 1,400)	0,567	-0,078 (0,893)
Flujo espiratorio máximo	-0,011 (-0,058 a 0,036)	0,637	
Cooximetría	-0,237 (-1,041 a 0,566)	0,553	
Minutos de ejercicio diario	-0,017 (-0,203 a 0,167)	0,846	
*Ventilation score previo*			
Estatura	-0,367 (-2,908 a 2,172)	0,770	0,066 (0,168)
Flujo espiratorio máximo	0,055 (-0,055 a 0,166)	0,314	
Cooximetría	-0,232 (-2,106 a 1,640 )	0,802	
Minutos de ejercicio diario	0,443 (0,011 a 0,875)	0,044	
*Ventilation score posterior*			
Estatura	-0,230 (-2,219 a 1,759)	0,816	0,024 (0,306)
Flujo espiratorio máximo	-0,056 (-0,143 a 0,030)	0,193	
Cooximetría	-0,992 (-2,459 a 0,474)	0,179	
Minutos de ejercicio diario	-0,078 (-0,417 a 0,259)	0,640	

	*Regresión logística*		
*Variable*	*Estimador OR (IC95%)*	*p*	*R*^*2*^*ajustado (p)*
*Relación compresión/ventilación previa*			
Estatura	0,876 (0,734 a 1,024)	0,114	-
Flujo espiratorio máximo	0,998 (0,990 a 1,005)	0,707	
Cooximetría	0,980 (0,854 a 1,097)	0,74	
Minutos de ejercicio diario	0,997 (0,863 a 1,024)	0,853	
*Relación compresión/ventilación posterior*			
Estatura	0,928 (0,790 a 1,077)	0,334	-
Flujo espiratorio máximo	0,999 (0,993 a 1,006)	0,985	
Cooximetría	0,975 (0,853 a 1,091)	0,679	
Minutos de ejercicio diario	1,009 (0,983 a 1,037)	0,48	

β: coeficiente de regresión lineal; OR: *odds ratio*; IC95%: intervalo de confianza al 95%.
